# Good MRI images: to Gad or not to Gad?

**DOI:** 10.1007/s00467-007-0509-z

**Published:** 2007-09-01

**Authors:** Henning Steen, Vedat Schwenger

**Affiliations:** 1 Department of Cardiology, University Hospital Heidelberg, Im Neuenheimer Feld 410, 69120 Heidelberg, Germany; 2 Department of Nephrology, University Hospital Heidelberg, Im Neuenheimer Feld 162, 69120 Heidelberg, Germany; 3 Medizinische Universitätsklinik Heidelberg, Im Neuenheimer Feld 162, 69120 Heidelberg, Germany

**Keywords:** Magnetic resonance imaging, Gadolinium contrast agents, Side effects of gadolinium, Acute renal failure (ARF), Nephrogenic systemic fibrosis (NSF), End-stage renal disease (ESRD)

## Abstract

Gadolinium-based magnetic resonance imaging (MRI) contrast agents (Gad-CA) were formerly considered as alternatives to X-ray-employed iodinated media. Although originally thought to be nonnephrotoxic and proven to be nonhazardous in a healthy population, the Gad-CA safety issue is progressively more controversial in the high-risk group of end-stage renal disease (ESRD) patients. Recently, Gad-CAs have not only been blamed for harmless side effects such as dizziness or nausea but also for much more severe complications such as acute renal failure, pancreatitis, or even the development of so-called “nephrogenic systemic fibrosis” in patients with renal failure, culminating in the prohibition of gadodiamide (Omniscan) administration in ESRD patients and, due to renal-organ immaturity, in newborns and infants up to 1 year old. This editorial is written to give insights into the molecular structure of Gad-CAs as well as into the potential biochemical pathomechanisms underlying the aforementioned severe clinical manifestations. Furthermore, a review about the latest literature on Gad-CA nephrotoxicity is provided. Potential risk factors are mentioned and strategies to avoid deterioration of renal function are presented. Cases with Gad-CA-associated adverse events should be adequately documented and reported appropriately. MRI professionals should collaborate closely with their colleagues from other medical specialties to identify patients with adverse events.

As nephrologists and colleagues working with magnetic resonance imaging (MRI), we generally take for granted the relative safety of gadolinium contrast agents (Gad-CA) in terms of their nonadverse effects on our patients. On 7 February 2007, the medical community was informed that gadodiamide (Omniscan) is now not only contraindicated in patients with end-stage renal disease (ESRD), but also in pre- or postoperative liver transplantation patients and, due to their renal-organ immaturity, in newborns and infants up to 1 year old. This change in policy was motivated by recent reports of a possible link between the use of gadodiamide and the development of so-called “nephrogenic systemic fibrosis” (NSF) [1–4] in patients with renal failure. This discovery came as quite a surprise to the nephrology and radiology communities.

A glimpse into medical literature reveals approximately 200 cases of NSF, previously known as nephrogenic fibrosing dermopathy (NFD) [1–6]. One pediatric case is published in this issue [7]. NSF is characterized clinically by the thickening, induration, and hardening of the skin in the distal extremities, followed by the skin of the trunk but usually omitting the face [8]. The diagnosis of NSF is confirmed by skin biopsy. Characteristically, histopathologic features such as thickened collagen bundles with surrounding clefts, mucin deposition, and proliferation of fibroblasts and elastic fibers are present, whereas signs of inflammation are absent, which makes this disorder a distinct entity [2, 8]. Grobner [8] was the first to report that five of nine gadodiamide-exposed hemodialysis patients developed NSF within 2–4 weeks after contrast-dye exposure. A systemic process is assumed in which circulating fibrocytes of bone marrow origin are aberrantly recruited to various body sites, including the skin, likely triggered or exacerbated by Gad-induced endothelial damage [9]. Following this report, Marckmann [10] also described 13 cases of NSF in patients exposed to gadodiamide, progressing in more than 50% of the cases to severe disability and even one patient death.

To date, there are eight Gad-based contrast media available in Europe: gadobenate-dimeglumine (Multihance, Bracco Diagnostics, Inc.), gadobutrol (Gadovist, Schering), gadodiamide (Omniscan, GE Healthcare), gadofosveset (Vasovist, Schering), gadopentetate-dimeglumine (Magnevist, Schering), gadoteric acid (Dotarem, Guerbet), gadoteridol (ProHance, Bracco Diagnostics Inc.), and gadoxetic acid disodium (Primovist, Schering). In each product, the Gad ion is chelated to various anion groups to form Gad salts [11].

The mechanism by which some Gad-CAs might trigger NSF is still unresolved; however, several theories have been proposed. Gad-CAs have different molecular structures that affect their biochemical properties. For example, Omniscan and OptiMARK have a linear and uncharged molecular configuration with excess chelate, which seems to be more likely to release free gadolinium ions (Gd3+) into the body, a process called transmetallation. Linear and charged (e.g., Magnevist, MultiHance, Primovist, and Vasovist) as well as cyclic and uncharged (e.g., Gadovist and ProHance) Gad-CAs seem to be less likely to release free Gd3+ into the body. Dotarem, for example, has a molecular charge and a cyclical structure and is least known for transmetallation. However, the exact mechanisms by which free gadolinium ions can stimulate NSF is unknown and therefore under investigation. Most of the approximately 200 cases of NSF that were reviewed so far in the literature have been associated with the agents Gadodiamide (Omniscan) and Gadoversetamide (OptiMARK, Mallinckrodt), which is not licensed for use in the European Union (EU) but is available in the USA. A small number of cases have been associated with Gadopentetate Dimeglumine (Magnevist, Schering), and to date, no cases of NSF have been associated with other gadolinium-containing contrast agents.

These alarming reports give rise to a nagging question: Is this just the tip of the iceberg? Have clinicians overlooked other possible side effects of Gad? The widespread reliance on Gad due to its nontoxicity probably needs to be reexamined. These MRI contrast agents were originally introduced as alternatives to iodinated media; iodine contrast dyes have become the third most common cause of acute renal failure. In patients with preexisting renal insufficiency, the incidence was as high as 50% [6]. Although originally thought to be nonnephrotoxic, Gad-based contrast media have recently been implicated in various other critical incidents (apart from NSF) in patients with ESRD. Again, a glance at the literature reveals that colleagues have been studying Gad-contrast-induced nephrotoxicity (CIN) since the early 1990s. CIN is now defined by the European Society of Urogenital Radiology (ESUR) [12] as an increase in serum creatinine of more than 25%, or more than 0.5 mg/dl, occurring within 3 days after the intravascular administration of the contrast medium in the absence of an alternative etiology. Interestingly, the controversial results are mostly due to the differing definitions of nephrotoxicity, patient’s collectives, renal diseases, contrast agents, and contrast-agent concentrations that have been employed and published. Unfortunately, there are almost no published data on pediatric patients with renal impairment.

As early as 1992, Haustein et al. [13] reported that Gad-Diethylene triamine pentaacetic acid (DTPA) [0.1 mmol/kg body weight (bw)] was harmless in a study of 21 patients with chronic renal failure (creatinine clearance 34.5 ml/min). Ten years later, Rieger et al. [5] described only one episode of acute renal failure in 39 Gad-DTPA (0.35 mmol/kg bw) angiographic imaging procedures for 29 patients (59% of whom were diabetic) with renal insufficiency (mean serum creatinine: 3.6 mg/dl). However, recent reports paint a different picture. Erley et al. [14] recorded a significant decline of renal function in 11 of 21 patients with severe renal impairment (mean creatinine clearance 31 ml/min) after gadobutrol (>0.5 mmol/kg) digital subtraction angiography. In a larger study, Sam et al. [15] comprehensively investigated 195 patients with chronic renal insufficiency (creatinine clearance 38 ml/min) who were subjected to Gad angiography (0.25 mmol/kg bw or more) and found that acute renal failure developed in seven patients (3.5%). Serum creatinine returned to baseline levels within 4–12 days in five patients, but ESRD developed in two patients. In our own collective of 50 patients with pre-ESRD or ESRD referred for cardiovascular MRI dobutamine stress echo test, and myocardial perfusion/viability, we observed a marked systemic inflammatory response signalled by increased inflammatory markers and fever up to 40°C and partial hypereosinophilia in seven patients 24 h after Gad-DTPA administration (0.3 mmol/kg bw). Acute renal failure was experienced in two patients, one of whom did not recover.

Do we know what mechanisms lead to renal impairment or systemic reaction after gad exposure? Unfortunately, due to sparse data on the renal lesions associated with this condition, very little is known about the mechanism of Gad nephrotoxicity. The nephrotoxic effects of iodinated contrast agents, which have been studied more intensely, are multifactorial, including a vasoconstrictive effect leading to hypoxic or ischemic tubular cell injury and a direct tubulotoxicity mediated by the generation of reactive oxygen species [12]. As Gad-based and iodinated contrast agents share the same pharmacodynamics [12, 15] and their nephrotoxic effects are often clinically similar, they may cause renal damage through the same mechanisms. The mechanism of the severe inflammatory reaction is akin to a massive allergic reaction, but the exact pathomechanism is poorly understood. Initiation of dialysis may promptly relieve the symptoms. Recently, Akgun [16] published a renal biopsy study documenting a reversible acute tubular cell injury in which patchy tubular cell necrosis and degeneration along with mild interstitial edema and inflammation were observed. The patient had been doubly exposed to Gad in the forms of Gad-DTPA and gadopentetate. No significant glomerular or vascular changes were observed.

Gad chelates are distributed in the extracellular space and are eliminated almost exclusively by the kidney through glomerular filtration. Renal failure impairs but maintains Gad excretion without resorting to a nonrenal route. For example, gadodiamide half-life is 1.3 h, 34.3 h, and 52.7 h in healthy patients, patients with ESRD, and peritoneal dialysis patients, respectively [17]. This extremely long half-life raises the question of how to handle pediatric patients on peritoneal dialysis (PD). Should they be hemodialyzed after Gad exposure, or should Gad exposure be completely avoided in PD patients? Further data are necessary to determine an optimal form of treatment.

What other potential systemic Gad effects could be responsible for the severe adverse events? Gadodiamide, for example, leaves two to four times more Gad in the bone than other contrast agents in patients with normal renal function [10, 12]. Because of the longer half-life of Gad-based contrast media in patients with ESRD, it could be speculated that Gad liberation might be causing systemic effects.

Taken together, these issues confront us with the task of correctly identifying and preparing our renal failure patients—and especially pediatric patients—with ESRD who were frequently treated with PD. At the same time, we must protect them from an unjustified risk caused by the possibility of adverse Gad-associated events. Raised serum creatinine levels (particularly secondary to diabetic nephropathy), dehydration, congestive heart failure, an age of over 70 years, and concurrent administration of nephrotoxic drugs (nonsteroidal anti-inflammatory drugs) are all considered potential risk factors by the guidelines [12]. Consequently, patients undergoing a diagnostic MRI scan with potential Gad exposure should be well hydrated, low- or iso-osmolar contrast media should be used, and nephrotoxic drugs should have been stopped for at least 24 h. Alternatively, other imaging techniques that do not require the administration of Gad should be taken into consideration. All in all, the less Gad, the better! In patients on continuous ambulatory PD (CAPD) and severely reduced renal function, one should avoid Gad doses exceeding 0.3 mmol/ kg bw [12].

The treatment of Gad-induced nephropathy begins with recognizing the condition. Therefore, in high-risk patients, the measurement of serum creatinine (a threshold of 1.5 mg/dl may be the ideal level for Gad agents, as is common with iodinated contrast agents) between the second and fourth day post-MRI will identify the nonaffected patients. Additionally, patients should not be reexposed to Gad before the kidney function has returned to its previous state. If Gad contrast media has to be administered again, the patients should at least be adequately hydrated and nephrotoxic agents should be stopped in due time.

Prompt hemodialysis within the first several hours after MRI may be recommended for quick removal of the Gad agent from the bloodstream. MRI professionals should collaborate closely with their colleagues from other medical specialties in the search to identify patients with Gad-associated adverse events. Cases with a positive history of exposure to Gad and adverse events should be adequately documented and reported through the appropriate channels. More data are required to clarify whether these complications are limited to one specific MRI contrast agent or might also occur with other types of Gad contrast media.

In conclusion, compelling evidence challenges the safety record of Gad contrast agents with respect to the conditions for which they were rather indiscriminately used in the past. We have to admit that the “carefree” days—when many of us thought that we at last had one imaging modality wherein contrast agents could be used nearly risk-free in renal-failure patients—are gone.
